# Large three-dimensional cell constructs for tissue engineering

**DOI:** 10.1080/14686996.2021.1945899

**Published:** 2021-08-11

**Authors:** Jun-Ichi Sasaki, Gabriela L Abe, Aonan Li, Takuya Matsumoto, Satoshi Imazato

**Affiliations:** a Department of Biomaterials Science, Osaka University Graduate School of Dentistry, Suita, Japan; b Department of Biomaterials, Okayama University Graduate School of Medicine, Dentistry and Pharmaceutical Sciences, Okayama, Japan; c Department of Advanced Functional Materials Science, Osaka University Graduate School of Dentistry, Suita, Japan

**Keywords:** Cell-based biomaterial, biomimetic material, organoid, in vitro tissue engineering, thermo-responsive hydrogel, bone regeneration, dental pulp regeneration, 30 Bio-inspired and biomedical materials; 211 Scaffold / Tissue engineering/Drug delivery

## Abstract

Much research has been conducted on fabricating biomimetic biomaterials in vitro. Tissue engineering approaches are often conducted by combining cells, scaffolds, and growth factors. However, the degradation rate of scaffolds is difficult to control and the degradation byproducts occasionally limit tissue regeneration. To overcome these issues, we have developed a novel system using a thermo-responsive hydrogel that forms scaffold-free, three-dimensional (3D) cell constructs with arbitrary size and morphology. 3D cell constructs prepared using bone marrow-derived stromal stem cells (BMSCs) exhibited self-organizing ability and formed bone-like tissue with endochondral ossification. Endothelial cells were then introduced into the BMSC construct and a vessel-like structure was formed within the constructs. Additionally, the bone formation ability was promoted by endothelial cells and cell constructs could be freeze-dried to improve their clinical application. A pre-treatment with specific protein protectant allowed for the fabrication of novel bone substitutes composed only of cells. This 3D cell construct technology using thermo-responsive hydrogels was then applied to other cell species. Cell constructs composed of dental pulp stem cells were fabricated, and the resulting construct regenerated pulp-like tissue within a human pulpless tooth. In this review, we demonstrate the approaches for the in vitro fabrication of bone and dental pulp-like tissue using thermo-responsive hydrogels and their potential applications.

## Graphical abstract

**Figure:**
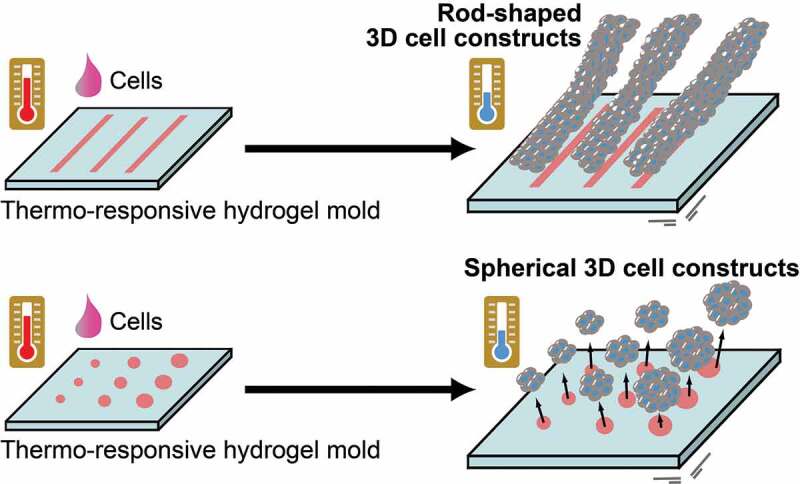

## 1. Introduction

Tissue engineering is a promising technology to regenerate living tissue in vitro and in vivo [1–3]. Various approaches using composites of cells, growth factors, and scaffolds have been fabricated for a variety of tissues or organs [4,5]. Specifically, skeletal muscle-like tissue was formed by the combination of type-I collagen and myoblasts, and myotubes and a sarcomere structure were observed within 3 weeks of culture [6]. For cartilage regeneration, a biomimetic scaffold was developed by combining artificial peptides and biodegradable polymers [7]. The chondrocytes embedded in the copolymer were capable of forming ectopic cartilage in mice.

Despite the progress of tissue engineering technologies, autologous and allogeneic transplantations are still the most common treatments for tissue defects [8–10]. There are several reasons why some cells and growth factors used in tissue engineering might show rapid clearance and low biosafety [11–14]. Furthermore, there are issues with scaffolds, such as the degradation rate being difficult to control and the degradation byproducts of the scaffolds inhibiting tissue regeneration [15–17]. In bone tissue engineering, organic and inorganic scaffolds, including calcium phosphate, collagen, poly (lactic-co-glycolic acid), and metals, are used as bone substitutes [18–21]; however, there is still no gold standard biomaterial.

We hypothesized that the problems described above can be resolved by a technology that enables the fabrication of a three-dimensional (3D) biomaterial comprising only cells, without a scaffold. In addition, the self-organization ability that cells originally possess should be promoted by the environment established by the cells comprising a 3D cell construct. Therefore, biomimetic materials, showing similar composition and structure to living tissue, may be fabricated using a 3D cell construct in vitro.

**Figure 1.**
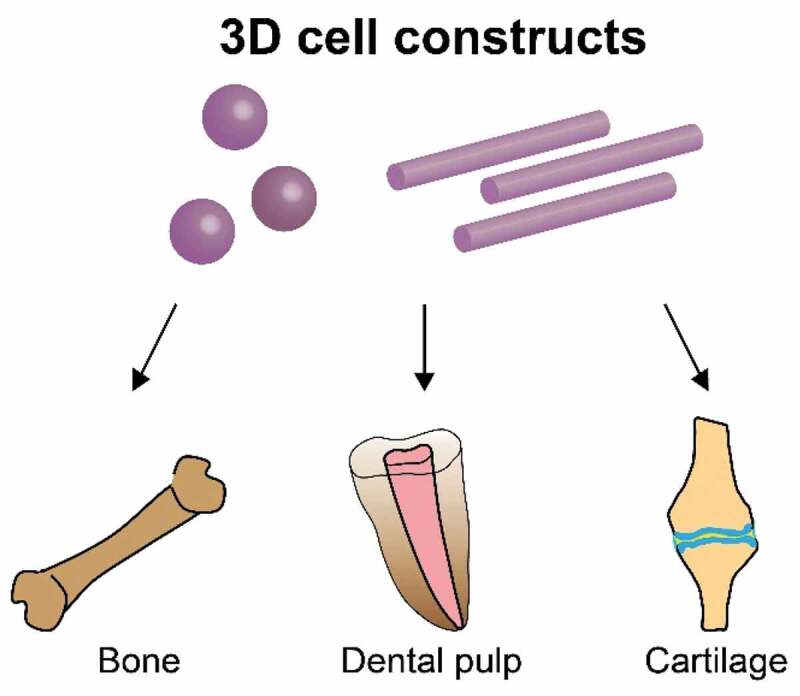
3D cell constructs can be fabricated in controlled morphologies and applied to regenerate a variety of specialized tissues such as bone, dental pulp, and cartilage

Several protocols have reported the fabrication of cell aggregates, referred to as spheroids [22,23], that are considered to be applicable to drug development, embryology, and regenerative medicine. Cell spheroids can be formed by various methods such as the hanging drop method [24,25], a non-adherent culture substratum [26,27], and rotating equipment [28,29]. However, these methods cannot produce large spheroids and it is also difficult to control the morphology of the cell aggregates. Therefore, a novel technology must be established to control the size and morphology of a 3D cell construct for application to regenerative medicine. In this review, we present the approach to fabricate large 3D cell constructs with a controlled morphology and their application for tissue engineering (Figure 1).

## 2. Fabrication of 3D cell constructs using a thermo-responsive hydrogel

### 2.1. Development of thermo-responsive hydrogel

Stimuli-responsive polymers, intelligent materials, change their microstructure according to the surrounding environment including temperature [30,31], pH [32,33], luminance [34,35], and magnetic and electric field fluctuations [36–39]. The hydrophilicity of a thermo-responsive polymer changes according to the temperature. For example, poly-*N*-isopropylacrylamide (pNIPAAm), which has a low critical solution temperature (LCST) [40,41], was used for preparing two-dimensional (2D) cell aggregates, referred to as cell sheets [42,43]. We hypothesized that if the cell sheet was formed on the pNIPAAm-coated 2D surface, scaffold-free 3D cell constructs comprised only cells that could be fabricated using 3D-rendered pNIPAAm. Therefore, the pNIPAAm hydrogel was evaluated as a mold for forming 3D cell constructs with a variety of sizes and morphologies.

**Figure 2.**
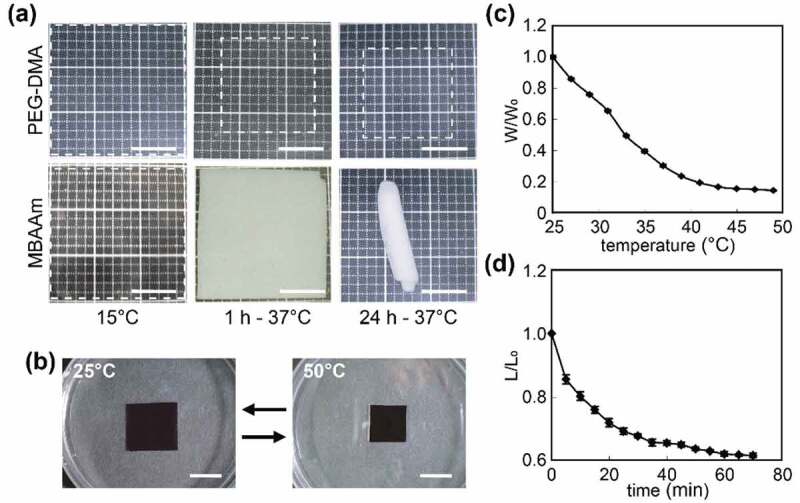
Characteristics of the poly-*N*-isopropylacrylamide (pNIPAAm) hydrogel. (a) The pNIPAAm hydrogel cross-linked with polyethylene glycol dimethacrylate (PEG-DMA) and *N,N’*-methylenebisacrylamide (MBAAm). Dashed squares indicate the gel shape. (b) Macroscopic images, (c) deswelling ratio, and (d) size alteration of the pNIPAAm hydrogel cross-linked with PEG-DMA depending on temperature (25–50°C). Scale bars: 1 mm. Reproduced with permission from Sasaki et al. [44]. Copyright 2010, Mary Ann Liebert, Inc

**Figure 3.**
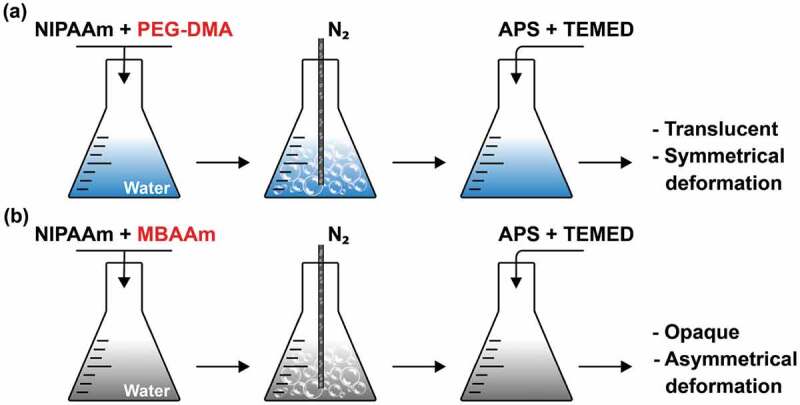
Fabrication of pNIPAAm thermo-responsive hydrogels. *N*-isopropylacrylamide (NIPAAm) crystals were dissolved in ultra-pure water and either (a) polyethylene glycol dimethacrylate (PEG-DMA) or (b) *N,N’*-methylenebisacrylamide (MBAAm) were added as cross-linking agents. To remove oxygen from the solution, an inert gas (nitrogen) was introduced at a constant flow. Then, ammonium persulfate (APS) and *N,N,N’,N’*-tetramethylethylenediamine (TEMED) were added as polymerization initiator and accelerator, respectively. PEG-DMA (a) was chosen as the appropriate cross-linking agent, considering the intended application of pNIPAAm gels

Polyethylene glycol dimethacrylate (PEG-DMA) and *N,N’*-methylenebisacrylamide (MBAAm) were tested as suitable cross-linking agents for pNIPAAm [44]. A pNIPAAm gel cross-linked with PEG-DMA showed high transparency at any temperature; however, a hydrogel prepared with MBAAm changed its color from transparent to opaque white at temperatures greater than 25°C (Figure 2a). In addition, the MBAAm-linking hydrogel rolled up with increasing temperature. The PEG-DMA cross-linked hydrogel did not show a clear LCST, though gradually and reversibly increased and decreased in volume with decreasing or increasing temperature, respectively (Figure 2(b,c,d)). Wettability analysis revealed that PEG-DMA cross-linked pNIPAAm demonstrated high hydrophilicity, which is favorable to detaching the cells at any temperature. Thus, pNIPAAm gels prepared by PEG-DMA were suitable for fabricating 3D cell constructs (Figure 3).

### 2.2. Fabrication of 2D/3D cell constructs using pNIPAAm gel

The cells were cultured on a plate of pNIPAAm gel to fabricate the 2D cell sheet [44]. In that study, pNIPAAm gels were coated by fibronectin to improve the cell adherent property of the gels. As a result, the pNIPAAm gels showed no cytotoxicity and supported cell proliferation. Interestingly, confluent cells on the gel detached as a 2D cell sheet by the expansion of gel, which was induced by the increasing temperature. Furthermore, immunofluorescence staining revealed that fibronectin was deposited on the cell sheet and not on the pNIPAAm gel; therefore, the pNIPAAm gel released the cell sheet with extracellular matrices (ECMs).

**Figure 4.**
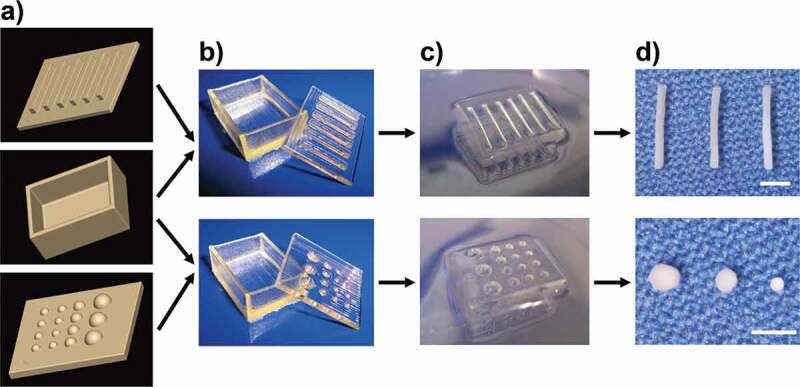
Fabrication of 3D cell constructs using pNIPAAm gels. (a) Solid polymer containers generated using 3D computer-aided design software. (b) Polymer containers manufactured by 3D printing system. (c) pNIPAAm gel molds with grooves (top) and dimples (bottom) on the surface. (d) 3D cell constructs with a variety of morphologies. Scale bars: 2 mm. Reproduced with permission from Sasaki et al. [44]. Copyright 2010, Mary Ann Liebert, Inc

Compared with the surface of 2D culture substrate, 3D gel is advantageous for generating 3D cell constructs with a variety of sizes and morphologies because the gel material is easily molded during sol–gel processing. For molding the gel, we designed solid polymer containers using 3D computer-aided design software and a 3D printing system (Figure 4(a,b)). Thermo-responsive pNIPAAm hydrogels with dimples and grooves were obtained by the gelation of the NIPAAm solution within these containers for 8 hours at 4°C (Figure 4c). The pNIPAAm gels were washed with phosphate buffered saline (PBS) for longer than 48 hours, then sterilized in 70% ethanol at 4°C until use. Osteoblast-like MC3T3-E1 cells were seeded on the channels of the pNIPAAm gel and cultured for 24 hours at 37°C, and the 3D cell constructs were finally harvested from the gel by decreasing the surrounding temperature, inducing the gel to expand and release the constructs (Figure 4d).

**Figure 5.**
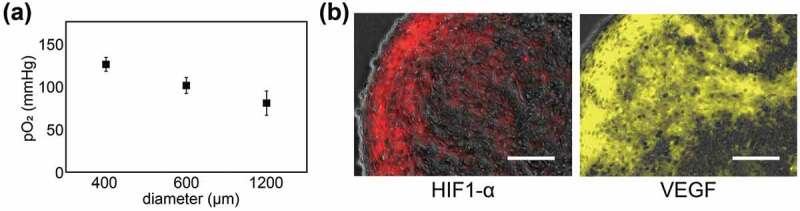
(a) Oxygen tension inside cell constructs with different diameters. (b) The expressions of hypoxia inducible factor (HIF)-1α and vascular endothelial growth factor (VEGF) in the spherical cell construct. Scale bars: 100 μm. Reproduced with permission from Sasaki et al. [46]. Copyright 2012, The Royal Society of Chemistry

3D cell construct is a simple cell aggregation connected only with cell–cell adhesion; therefore, cells in the construct are exposed to a low oxygen and nutrient condition [45]. First, the oxygen gradient within a spherical cell construct was measured for characterization of the construct [46]. The concentration of oxygen at the center of the construct decreased with increasing diameter of the constructs (Figure 5a). Additionally, the expressions of hypoxia-inducible factor (HIF)-1α and vascular endothelial growth factor (VEGF), known as hypoxia markers [47–49], were detected in the cells in the outer layer of the construct (Figure 5b). As a result, cells within the cell constructs experience chronic hypoxia and nutrient-poor environment.

## 3. Application of 3D cell constructs for bone regenerative biomaterials

A variety of bone substitutes based on calcium-containing compounds have been applied to bone regenerative medicine [50–53]. These biomaterials use the osteoconductivity of calcium phosphate [54]; however, bioactive components, including type-I collagen and growth factors, are not fully exploited for bone regeneration. A long time is needed to regenerate the bone tissue because bone substitutes require vessel infiltration, biodegradation, and replacement with bone tissue. Indeed, there are no biomaterials comparable to autologous bone, which contains inorganic components and bone-related proteins. Bone substitutes comprising cells and cell-derived ECMs are promising biomaterials for bone regeneration; therefore, 3D cell constructs may be useful for fabricating bone-like tissue in vitro.

### 3.1. Osteogenic differentiation of 3D cell constructs

**Figure 6.**
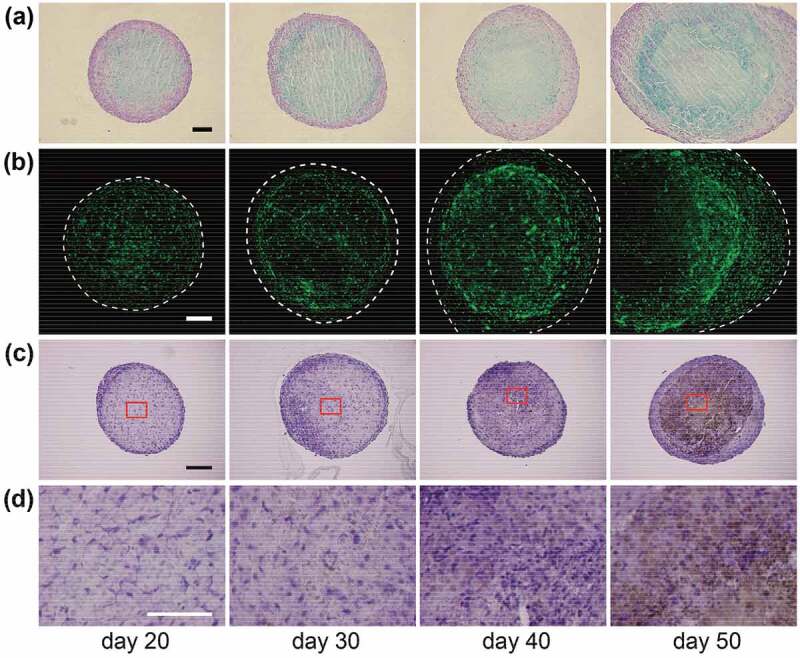
Osteoblastic differentiation of BMSC constructs. (a) Cartilaginous matrices were detected by (a) Alcian blue staining and (b) type II collagen immunostaining. (c) The mineralized matrix was observed by von Kossa staining and (d) the magnified images are indicated by red lines in (c). Scale bars: (a–c) 100 μm and (d) 50 μm. Reproduced with permission from Sasaki et al. [46]. Copyright 2012, The Royal Society of Chemistry

A hypoxia condition promotes the chondrogenic differentiation of bone marrow-derived stromal stem cells (BMSCs) [55–57]. BMSCs, one of the somatic stem cells, possess stemness which is capable to differentiate into mesenchymal cells including osteoblasts, chondrocytes, myoblasts, and fibroblasts. Cell constructs were prepared using only BMSCs and cultured by osteogenic differentiation medium containing ascorbic acid, β-glycerophosphate, and dexamethasone [58,59] for up to 50 days [46]. Interestingly, histological evaluation of the BMSC constructs revealed that cartilage matrices (e.g. type II collagen and chondromodulin-I [60,61]) were deposited in the middle layer of the construct (Figure 6(a,b)). In addition, the cells in the middle layer resembled the morphology of hypertrophic chondrocytes. These results indicated that BMSCs in the middle layer of the construct differentiated into chondrocytes.

The deposition of bone matrices was observed in the BMSC construct, with mineralized matrix formed in the center of the construct (Figure 6(c,d)). Based on X-ray diffraction and energy-dispersive X-ray spectroscopy analyses, the distribution and ratio of calcium and phosphate components were compatible with those of apatite crystals.

Collectively, BMSC constructs are capable of forming cartilage matrices in the middle layer and mineralized matrices in the center part. BMSC constructs possess the self-organizing ability and structure of bone-like tissue with endochondral ossification [46]. These characteristics of the BMSC construct are considered to be applicable to biomaterials for bone regeneration.

### 3.2. Vasculature formation in 3D cell constructs

Vasculature formation into cell-based biomaterials is essential for improving the cell viability and activity [62–65]. Indeed, some cells die in the center of the construct because of hypoxia and malnutrition. Human umbilical vein endothelial cells (HUVECs) were used to form the vasculature within the cell constructs [66]. The suspension of HUVECs was mixed with BMSCs at 1–5% and BMSC/HUVEC constructs (99:1–99:5) were fabricated using the pNIPAAm gel system. The 3D cell construct comprising only BMSCs was referred to as 100:0.

**Figure 7.**
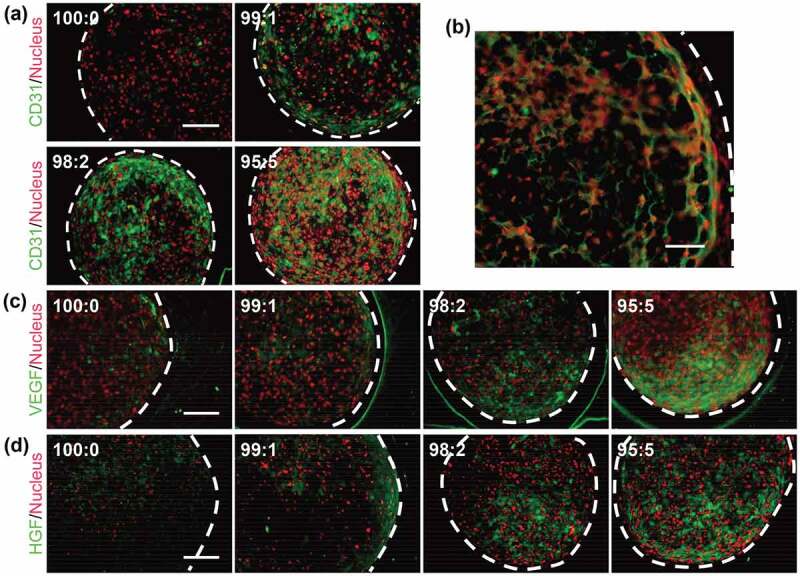
Immunofluorescence stained images of BMSC/HUVEC 3D constructs. (a) Stained area of HUVECs (CD31-positive cells; green), which was more diffuse with the increasing ratio of HUVECs. The BMSCs only stained positive for Hoechst33342 (nucleus; red). (b) Representative magnified image of a BMSC/HUVEC (99:1) construct showing that HUVECs formed a reticulated structure within the 3D cell construct. (c) VEGF and (d) HGF angiogenic factors were diffused within each cell construct. White dotted lines indicate outline of cell construct. Scale bars: (a, c, d) 100 μm and (b) 50 μm. Reproduced with permission from Sasaki et al. 2015 [66]

The histological observation revealed that live cells within the cell constructs increased with increasing ratio of HUVECs [66]. In addition, HUVECs were alive and formed a vessel-like reticular structure within the constructs (Figure 7(a,b)). Noting that HUVECs were unable to survive in the osteogenic medium used in that study, BMSCs were considered to support the viability of HUVECs in the BMSC/HUVEC construct. The expression of the angiogenic growth factors was investigated in the constructs; the deposition of VEGF and hepatocyte growth factor (HGF) was greater in BMSC/HUVEC constructs than the BMSC only construct (Figure 7(c,d)). These results suggested that the production of angiogenic growth factors was promoted by the increase in survival rate of BMSCs due to the vasculature formation of HUVECs. Additionally, these growth factors could help the survival of HUVECs in the osteogenic medium.

Regarding the bone formation ability, a BMSC/HUVEC constructs with high HUVECs ratio promoted the deposition of mineralized matrices compared with the BMSC construct [66]. Cell–cell communication between the BMSCs and HUVECs with secreted cytokines, such as bone morphogenetic proteins, might have enhanced the osteogenesis of BMSCs [67]. Furthermore, the alkaline phosphatase (ALP) activity, calcium content, and hardness also increased with increasing ratio of HUVECs in the constructs. This study indicated that BMSCs and HUVECs had biological mutualism within the cell constructs and promoted bone formation ability, further demonstrating the fabrication of bone-like biomimetic tissue in vitro.)

### 3.3. Freeze-dry processing of 3D cell constructs

Cell-based biomaterials are considered to have problems in tumorigenicity, preservability, and susceptibility to infection [68–70]. To resolve these issues, decellularization treatment is often conducted by detergent, high-pressure, or freeze-drying of the biomaterials [71–73]. We focused on the freeze-dry processing that has been applied to medical products and food industry because this technology also improves the operability and stability of cell-based biomaterials [74]. During the freeze-dry processing of cell-based biomaterials, it is also important to inhibit the denaturation and deactivation of proteins [75,76]. Protein protectants, which are capable of forming a hydrogen bond with proteins and suppressing deformation of the protein conformation, are used for manufacturing of pharmaceutical products and research materials [77–79]. Non-reducing disaccharides (e.g. sucrose and trehalose) can be used as protein protectants, and sucrose has been the most studied protectant for freeze-dry processing [80–82].

**Figure 8.**
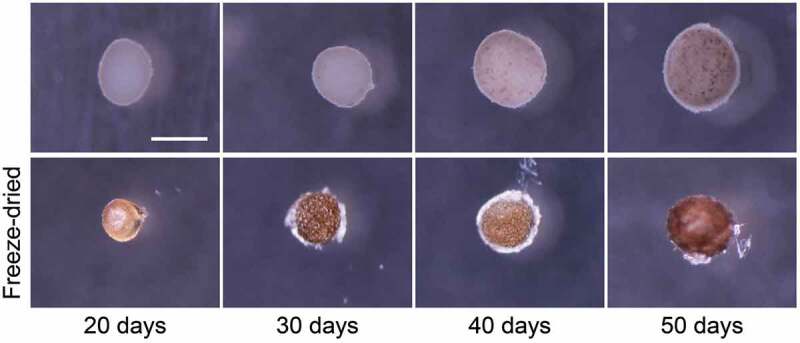
Freeze-dry processing of 3D BMSC constructs. Images of the cell constructs before (top) and after (bottom) freeze drying. Scale bar: 500 μm. Reproduced with permission from Sasaki et al. [83]. Copyright 2019, Wiley Periodicals, Inc

To establish the freeze-dry protocol for 3D cell constructs as a biomimetic bone tissue, the BMSC constructs were pretreated with or without sucrose [83]. The experiments showed that the size of the BMSC construct decreased and the morphology was maintained (Figure 8). Additionally, the PBS treatment without sucrose significantly reduced the ALP activity in the freeze-dried cell constructs compared with the sucrose treated constructs. In contrast, 10 wt% sucrose in PBS maintained the ALP activity in the constructs. These results indicated that the freeze-dry processing of 3D cell constructs with suitable protein protectants enabled the fabrication of a novel bone substitute comprising only cells [83].

## 4. Application of 3D cell constructs for dental pulp regeneration

Dental pulp assumes a variety of roles including maintenance of tooth homeostasis, pain transmission, and regeneration of dentin [84,85]. Generally, dental pulp is extracted in the case of irreversible pulpitis and pulp necrosis, then the root canal is filled with a rubber-based material [86,87]. However, pulpless teeth lose their natural biological defense, increasing the risk of serious caries, apical periodontitis, and ultimately tooth loss [88,89]. Specifically, the hazard ratio for tooth loss increases 7.4-fold for pulpless molars and 1.8-fold for pulpless anterior teeth and premolars relative to their pulp-conserved counterparts [90]. Thus, dental pulp regeneration can recover the function of teeth and improve the prognosis of a pulpless tooth. 3D cell constructs were fabricated using dental pulp stem cells (DPSCs) and their ability to regenerate dental pulp was evaluated [91].

### 4.1. Fabrication of 3D cell constructs comprising DPSCs

**Figure 9.**
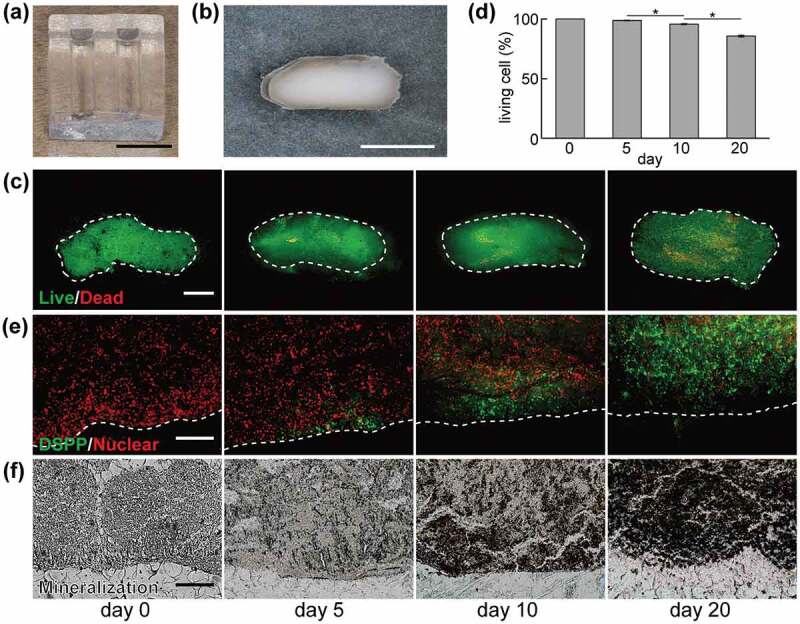
Fabrication and characteristics of the dental pulp stem cell (DPSC) constructs. (a) The pNIPAAm gel for forming the DPSC construct. (b) Stereoscopic image of the DPSC construct. (c) Images of live/dead staining and (d) semiquantitative analysis of the living cells. Each bar represents the mean ± SD. *n* = 4. *P < 0.05. The panels show (e) dentin sialophosphoprotein (DSPP) immunofluorescence staining, and (f) von Kossa staining for mineral deposition. Dotted lines indicate the outermost surface of the cell construct. Scale bars: (a) 10 mm, (b) 5 mm, (c) 500 μm, and (e, f) 100 μm. Reproduced with permission from Itoh et al. [91]. Copyright 2018, International & American Associations for Dental Research

To use the DPSC construct as a transplant, the size of cell constructs should be adjusted to the morphology of a root canal. Thus, we prepared pNIPAAm gel with large grooves (length, 12 mm; width, 3.0 mm; depth, 3.0 mm) (Figure 9a). In this study, the method for fabricating the DPSC construct was modified to use the cell sheet harvested by a cell scraper from a 100-mm culture dish. As a result, rod-shaped cell constructs with a major axis of 7 mm and short diameter of 3 mm, were successfully obtained from the pNIPAAm gel (Figure 9b). The DPSC viability in the constructs was >85%, even after 20 days of culture (Figure 9(c,d)).

### 4.2. In vitro self-organizing ability of DPSC constructs

DPSCs are capable of differentiating into odontoblasts by culturing in a mineralizing environment [92,93]. Additionally, cell spheroids exhibit a self-organizing ability that is inherent to living tissue in the body [94,95]. Therefore, the self-organizing ability of DPSC constructs was examined by inducing odontoblastic differentiation [91]. Dentin sialophosphoprotein (DSPP), an odontoblastic differentiation marker [96], was localized at the outer layer of the DPSC constructs after 5 days of differentiation (Figure 9e). Mineralized matrices, one of the main components of dentin, were observed at the outer layer of the construct at 10 days of culture (Figure 9f). Thereafter, the deposition area of DSPP and mineralized ECMs increased from the surface to the center of the DPSC constructs at 20 days of culture. These results suggested that the differentiation state of the DPSCs in the outer layer and center part of a construct was temporary different.

The messenger ribonucleic acid (mRNA) expression of the DPSCs harvested from the outer and center part of the constructs was evaluated. The expression of *DSPP* significantly increased in the DPSCs in the outer layer of the constructs compared with the cells in the center part. Interestingly, the expression of *Nanog*, a stem cell marker [97], was higher in the DPSCs located at the center of construct. These results indicate that DPSCs in the outer layer of the constructs differentiated into odontoblasts, whereas the DPSCs in the center part maintained their stemness. Thus, the DPSC constructs possess a self-organizing ability, which facilitates the dental pulp regeneration in a pulpless tooth [91].

### 4.3. In vivo dental pulp regeneration using DPSC constructs

To assess the pulp regeneration ability, a DPSC construct was packed into a human pulpless tooth, the therapeutic environment was simulated, and then the tooth was implanted into an immunodeficient mouse [91].

**Figure 10.**
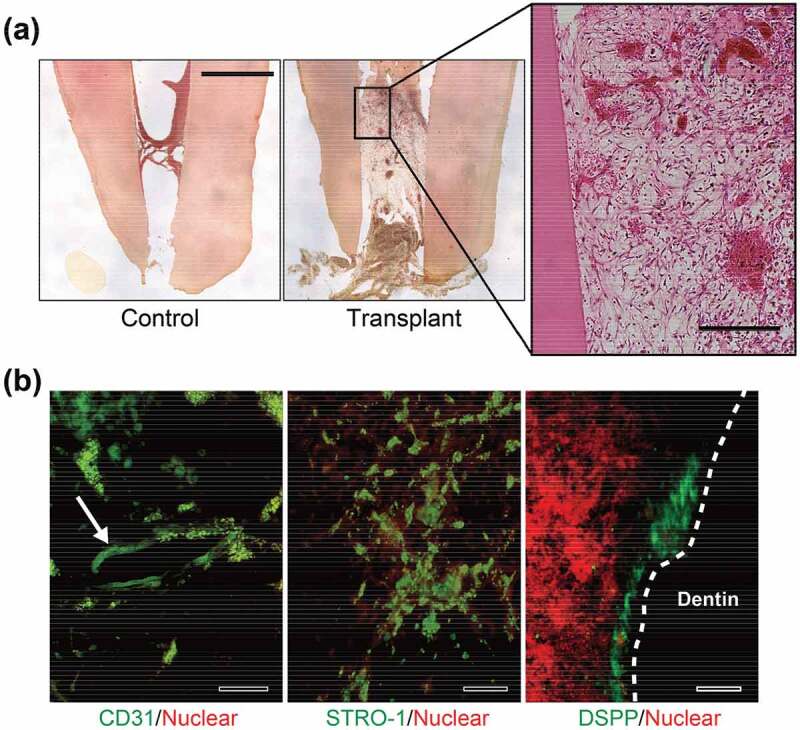
Dental pulp regeneration with 3D DPSC constructs. (a) Hematoxylin–eosin staining of DPSC construct-transplanted tooth root. The right panel shows magnified images of the box-enclosed area from the transplanted specimen. The control specimen was a human tooth root without a DPSC construct. (b) Immunofluorescence staining for CD31, STRO-1, and DSPP was performed in a DPSC construct-transplanted human tooth root. The arrow indicates the vessel-like formation comprised of human CD31-positive cells with host blood cells. The dotted lines indicate the dentin surface of the tooth root canal. Scale bars: (a) 5 mm (1 mm in magnified image) and (b) 100 μm. Reproduced with permission from Itoh et al. [91]. Copyright 2018, International & American Associations for Dental Research

Consequently, pulp-like tissue was formed within the DPSC construct transplanted root canal after 6 weeks of implantation; however, there was no tissue formed inside of a root canal without a cell construct (Figure 10a). Additionally, regenerated tissue contained luminal structures comprising human CD31-positive cells, suggesting that transplant DPSCs differentiated into endothelial cells and formed blood vessels (Figure 10b). Further histological evaluation revealed that STRO-1-positive stem cells were distributed in the regenerated tissue, and DSPP-positive odontoblast-like cells were localized at a site close to the dentin wall that had been implanted with the DPSC construct (Figure 10b).

The DPSC constructs allow the dental pulp tissue to regenerate in a pulpless tooth. Technology using DPSC constructs shows promise for achieving tailor-made pulp regeneration therapy that can be adjusted for each pulpless tooth [91].

## 5. Further application of 3D cell constructs

The technology for fabricating 3D cell constructs can be applied to other types of cells as well as the BMSCs and DPSCs described in this review [98,99]. Previously, we reported that 3D cell constructs were fabricated from the fibroblast-like L929 cells [98]. This study demonstrated a method for eliminating the dead cells from the cell construct using a syringe needle. Okawa et al. [99] prepared the cell constructs comprising only induced pluripotent stem (iPS) cells using pNIPAAm gel and induced osteogenic differentiation. The authors then showed that iPS cell constructs formed bone-like tissue in vitro. The iPS cells possess pluripotency [100], and as such, various tissue- and organ-like biomimetic biomaterials could be fabricated using 3D cell constructs comprising adequate stem cells.

The term organoid is interpreted as a 3D artificial organ produced in vitro [101–103]. Bone- and dental pulp-like tissues fabricated from 3D cell constructs are also categorized into the organoids. Recently, there has been an increase in organoid research and technologies using laminated cell sheets and a 3D bioprinter was established for in vitro tissue engineering [104–107]. Notably, the advantages of 3D cell constructs are 1) that they are easy to control the size (>10 mm) and morphology, 2) their simple construction, 3) their high biosafety and biocompatibility without a scaffold, and 4) their ability to reproduce the biomimetic environment because of their self-organizing ability. Therefore, it is considered that organ-like biomaterials originating from a 3D cell construct are applicable to pharmaceutical science and developmental biology as well as regenerative medicine. Meanwhile, tissue defects can often involve extremely large areas and various specialized tissues at once. These conditions remain a challenge to cell construct application given the great number of cells necessary to promote defect reconstitution. Nonetheless, iPS cells may represent an unlimited source of stem cells, which could be adopted to fabricate cell constructs of extreme sizes. This idea would benefit from further investigation on size and morphology, utilizing iPS cells as resource, and improving on the suitability of 3D cell construct for applications.

3D cell constructs have simple structures immediately after fabrication, and the cells can die because of insufficient nutrition and oxygen diffusion. The cell viability of a cell-based material is important when used as a transplant [108]. In this review, we demonstrated that a vessel-like structure could be formed within the construct using endothelial cells. However, HUVEC endothelial cells used in the current study was an immortalized cell line, and unsuitable for use in regenerative medicine and tissue engineering. Recently, DPSCs [109,110] and iPS cells [111] have been demonstrated to differentiate into endothelial cells. With such stem cells, vascularized cell constructs showing high biosafety could be fabricated in vitro, and these vascularized biomimetic constructs are promising for developing large, complex tissue-like biomaterials.

## 6. Conclusions

In this review, we described the development of a thermo-responsive hydrogel, creation of 3D cell constructs, and in vitro fabrication of bone- and dental pulp-like tissues. In addition, we have presented the availability and future of the cell constructs. Thermo-responsive pNIPAAm hydrogel can be used to fabricate 3D cell constructs with a variety of cell types, controlled size, and morphology; therefore, the cell constructs are well suited for clinical and research applications.
